# Correlations of non-exercise activity thermogenesis to metabolic parameters in Japanese patients with type 2 diabetes

**DOI:** 10.1186/1758-5996-5-26

**Published:** 2013-05-27

**Authors:** Hidetaka Hamasaki, Hidekatsu Yanai, Shuichi Mishima, Tomoka Mineyama, Ritsuko Yamamoto-Honda, Masafumi Kakei, Osamu Ezaki, Mitsuhiko Noda

**Affiliations:** 1 Department of Internal Medicine, National Center for Global Health and Medicine Kohnodai Hospital, Chiba, Japan; 2 General Internal Medicine, Community Healthcare Studies, Jichi Medical University Graduate School, Tochigi, Japan; 3 Department of Diabetes and Metabolic Medicine, Center Hospital, National Center for Global Health and Medicine, Tokyo, Japan; 4 First Department of Comprehensive Medicine, Saitama Medical Center, Jichi Medical University School of Medicine, Saitama, Japan; 5 Department of Human Health and Design, Faculty of Human Life and Environmental Sciences, Showa Women’s University, Tokyo, Japan; 6 Department of Diabetes Research, Diabetes Research Center, National, Center for Global Health and Medicine, Tokyo, Japan

**Keywords:** Atherosclerosis, Insulin, Non-exercise activity thermogenesis, Obesity, Type 2 diabetes

## Abstract

**Background:**

Non-exercise activity thermogenesis (NEAT) is the energy expenditure due to physical activities besides active sports-like exercise and resistance training in daily life.

**Methods:**

We studied 45 subjects (22 women and 23 men) with type 2 diabetes who did not take any hypoglycemic, anti-hypertensive, or cholesterol-lowering agents and asked them about physical activity concerned with NEAT using an original questionnaire modified from a compendium of physical activities. We studied the association of the NEAT score to body weight, waist circumference, blood pressure, glucose and lipid metabolism, and arterial stiffness.

**Results:**

The NEAT score was negatively correlated with serum insulin levels (r = -0.42, *P* < 0.05) in all subjects. The NEAT score was also negatively correlated with waist circumference (r = -0.509, *P* < 0.05) and positively correlated with high-density lipoprotein-cholesterol levels (r = 0.494, *P* < 0.05) in women, and negatively associated with serum insulin levels (r = -0.732, p < 0.005), systolic (r = -0.482, *P* < 0.05) and diastolic blood pressure (r = -0.538, *P* < 0.05) in patients with abdominal obesity. Furthermore, the NEAT score was negatively associated with pulse wave velocity (r = -0.719, *P* < 0.005) in smokers.

**Conclusion:**

The study demonstrated that NEAT is associated with amelioration in insulin sensitivity, waist circumference, high-density lipoprotein-cholesterol, blood pressure and the marker for atherosclerosis in patients with type 2 diabetes.

## Background

Non-exercise activity thermogenesis (NEAT) is the energy expenditure due to physical activities besides active sports-like exercise and resistance training [1]. It includes various activities in daily life such as going to work, attending school, singing, dancing, washing clothes and cleaning floors [1].

Greater prevalence of cardiovascular diseases (CVD) risk factors in urban or suburban residents is explained by low physical activity [2,3], and a sedentary lifestyle is a major cardiovascular risk factor [4].

The promotion of physical activity is crucial in the management of type 2 diabetes. Systematic reviews suggest that aerobic exercise and resistance training improve glycemic control in patients with type 2 diabetes [5-9]. However, to our knowledge, there were no previous studies that investigate the association between NEAT and metabolic parameters including body mass index, waist circumference, blood pressure, glucose and lipids metabolism, in patients with type 2 diabetes.

We calculated the NEAT score with a questionnaire for evaluating physical activity habits concerned with NEAT, and studied how NEAT correlates with metabolic parameters in patients with type 2 diabetes.

## Methods

### Study population

The study protocol was approved by the Medical Ethics Committee of the National Center for Global Health and Medicine (reference number NCGM-G-1151). We studied 45 subjects (22 women and 23 men) who did not take any hypoglycemic agents including metformin, and also did not take anti-hypertensive and cholesterol-lowering agents. Information about medical history, smoking status and medication were obtained via an interview. The subjects studied were aged between 20 and 90 years old, and were all diagnosed as having type 2 diabetes according to the Japanese diagnostic criteria for type 2 diabetes without physical disability. The clinical, biochemical and physiological characteristics of the subjects studied are shown in Table 1.

**Table 1:** Clinical biochemical and physiological characteristics of subjects

	
Number of subjects	45
Age (years old)	59.9 ± 14.5
Sex (male/female)	23 / 22
Smoking (current smoker/ ex- and non-smoker	27 / 18
Body height (cm)	160.3 ± 9.0
Body weight (kg)	65.0 ± 18.7
Waist circumference (cm)	92.5 ± 13.7
Body mass index (kg/m^2^)	26.0 ± 5.9
Systolic blood pressure (mmHg)	130.7 ± 18.6
Diastolic blood pressure (mmHg)	78.6 ± 16.9
Plasma glucose (mg/dl)	192.3 ± 106.4
Hemoglobin A1c (%)	8.0 ± 2.0
Serum Insulin (μlU/ml	18.3 ± 20.6
Serum low density lipoprotein cholesterol (mg/dl)	130.9 ± 31.4
Serum triglyceride (mg/dl)	181.1 ± 95.1
Serum high-density lipoprotein cholesterol (mg/dl)	51.7 ± 13.9
Pulse wave velocity (cm/s)	1667 ± 417

### Waist circumference, blood pressure, and arterial stiffness

Waist circumference was measured from the navel with the subjects breathing out while standing. Blood pressure was measured with subjects in a seated position using an automatic sphygmomanometer (HEM-762, Omron Co., Ltd, Kyoto, Japan), while arterial stiffness was examined by measuring the brachial-ankle pulse wave velocity (baPWV) using a pulse pressure analyzer (model: BP-203RPE; Nihon Colin, Tokyo, Japan).

### Laboratory measurements

Plasma glucose was measured using an enzymatic method (Wako Pure Chemical Industries, Osaka, Japan). Serum insulin and hemoglobin A1c were measured by automated enzyme-linked immunosorbent assays (TOSOH, Tokyo, Japan) and high-performance liquid chromatography (TOSOH), respectively. Total cholesterol, triglyceride (TG), high-density lipoprotein-cholesterol (HDL-C) and low-density lipoprotein-cholesterol (LDL-C) were determined enzymatically, using commercially available kits, Tcho-l, TG-LH (Wako Pure Chemical Industries), Cholestest N HDL and Choletest LDL (Daiichi Pure Chemicals, Tokyo, Japan), respectively.

### Assessment of NEAT

We asked the subjects about their habits of physical activity concerned with NEAT using an original questionnaire modified from a compendium of physical activities [10]. (See the Additional file 1). We created the NEAT score by referring to an article by Ainsworth BE and colleagues [10]. Technicians at the Clinical Research Center of the National Center for Global Health and Medicine at Kohnodai Hospital asked participants at the Outpatient Clinic about their typical habitual activities, and we also asked the subjects how high a level of exercise they engage in, including workouts in gyms, etc. Subjects who engaged in active sports-like exercise and resistance training were excluded. We evaluated each questionnaire item with a score of 1 to 3 points in order of levels of habitual physical activity and then added up the scores to determine the NEAT score.

### Statistical analysis

Statistical analysis was performed using SPSS version 19 (IBM Co., Ltd, Chicago, USA). All values were expressed as the mean ± standard deviation (SD), except for sex and smoking status. Pearson's correlation coefficient was calculated in order to analyze the association of the NEAT score with clinical, biochemical and physiological data. *P* < 0.05 was considered to be statistically significant.

## Results

**Figure 1:**
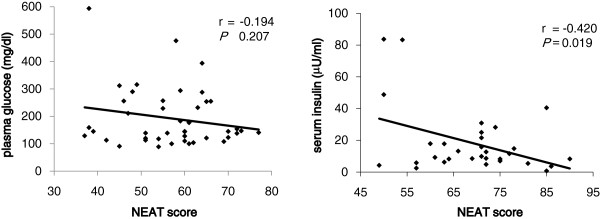
Correlation between the NEAT score and plasma glucose levels serum insulin levels in all subjects.

The mean ± SD of the NEAT score for all participants was 57.4 ± 11.8 (range: 37–80), and 56.9 ± 10.6 and 57.6 ± 10.1 for men and women, respectively. In all subjects studied, plasma glucose levels were not significantly associated with the NEAT score, but serum insulin levels were significantly and negatively correlated with the NEAT score (Figure 1).

**Figure 2:**
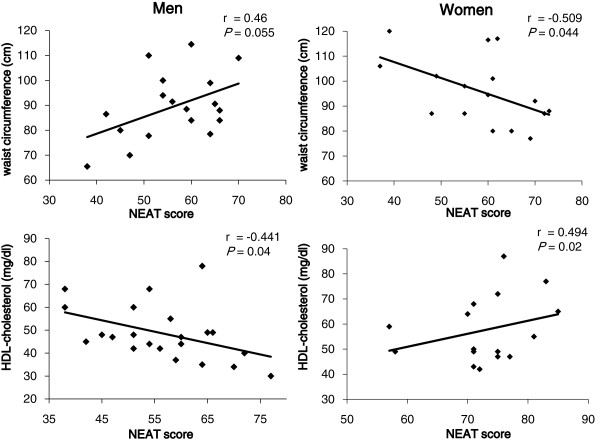
Correlation between the NEAT score and waist circumference and serum high-density lipoprotein (HDL)-cholesterol levels in men and women.

In all subjects, the NEAT score was not significantly correlated with waist circumference and HDL-C levels. In women, the NEAT score was significantly and negatively correlated with waist circumference and also was significantly and positively correlated with HDL-C levels (Figure 2). In men, we could not observe a significant association between the NEAT score and waist circumference. Furthermore, and interestingly, the NEAT score in men was significantly and negatively correlated with HDL-C levels.

**Figure 3:**
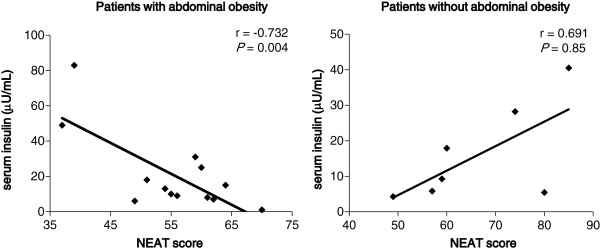
**Correlation between the NEAT score and serum insulin levels in patients with and without abdominal obesity.** Patients with abdominal obesity means male patients with waist circumference (WC) ≧ 85 cm and female patients with WC ≧ 90 cm, and patients without abdominal obesity means male patients with WC < 85 cm and female patients with WC < 90 cm.

We divided subjects into two groups by waist circumference (< or ≧ 90 cm for women and < or ≧ 85 cm for men, which is defined as abdominal obesity in the Japanese diagnostic criteria for metabolic syndrome [11]), because patients with abdominal obesity are at increased risk for CVD and hypertension [12]. In patients with abdominal obesity, the NEAT score was significantly and negatively associated with serum insulin levels (Figure 3). However, in patients without abdominal obesity, a significant association between the NEAT score and serum insulin levels was not observed.

**Figure 4:**
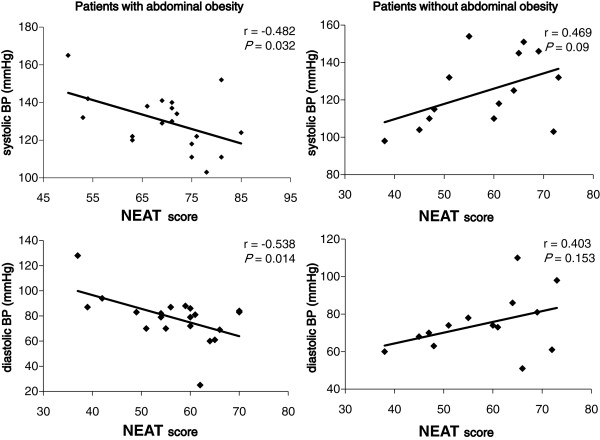
**Correlation between the NEAT score and systolic and diastolic blood pressure in patients with and without abdominal obesity.** Patients with abdominal obesity means male patients with waist circumference (WC) ≧ 85 cm and female patients with WC ≧ 90 cm, and patients without abdominal obesity means male patients with WC < 85 cm and female patients with WC < 90 cm.

Further, in patients with abdominal obesity, the NEAT score was significantly and negatively correlated with systolic and diastolic blood pressure (Figure 4), but in patients without abdominal obesity, we did not observe a significant association between the NEAT score and systolic and diastolic blood pressure.

**Figure 5:**
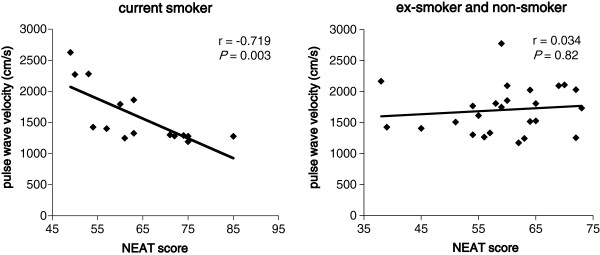
Correlation between the NEAT score and pulse wave velocity in current smokers and ex- and non-smokers.

Smoking has been reported to be associated with insulin resistance [13] and the development of type 2 diabetes [13,14]. Therefore, we divided subjects into two groups by smoking status. In current smokers, the NEAT score was significantly and negatively correlated with baPWV (Figure 5), but in ex- and non-smokers, there was no significant association between the NEAT score and PWV.

## Discussion

Previous studies have suggested that the amounts of physical activity such as exercise was significantly and negatively associated with serum insulin levels [15,16], meaning serum insulin levels were lower in active people when compared to sedentary people. In this study, the NEAT score was significantly and negatively associated with serum insulin levels, suggesting that NEAT is also associated with insulin sensitivity in addition to exercise. However, we have to mention that the correlation between NEAT and serum insulin may be largely dependent on the presence of three subjects with very high insulin and low NEAT. The NEAT score was not associated with plasma glucose levels in all participants, suggesting that the association between the NEAT score and glucose metabolism may be modest.

For all participants, the NEAT score was not correlated with waist circumference and HDL-C levels. In women with type 2 diabetes, the NEAT score was negatively associated with waist circumference and positively associated with HDL-C levels, indicating an association of NEAT with metabolic parameters in women. However, we could not observe this association in men. The study could not explain why this association of NEAT score to waist circumference and HDL-C in women was different from that in men, and we would have to perform further studies with a greater number of participants to explain the association of NEAT with waist circumference and HDL-C.

The metabolic syndrome, which is characterized by abdominal obesity, high TG, low HDL-C, elevated blood pressure, glucose intolerance and insulin resistance, has been identified as a clustering of risk factors for atherosclerotic diseases [17]. Insulin resistance has been reported to be significantly associated with abdominal obesity [17], and accumulated abdominal fat induces insulin resistance with the secretion of various cytokines and the reduced secretion of adiponectin [18,19]. The previous study showed that obese patients with both type 2 diabetes and hypertension had a significantly lower likelihood of adopting physical activity to control their weight than those with neither condition (6% versus 12%, *P* < 0.01) [20], suggesting that NEAT is more crucial for controlling body weight in obese patients with diabetes compared with those without diabetes. Our study showed that in patients with abdominal obesity, the NEAT score was negatively correlated with serum insulin levels, suggesting that NEAT is associated with insulin resistance in type 2 diabetic patients with abdominal obesity. NEAT has been reported to play an important role in controlling body weight in patients with obesity [21,22], which is supported by our result. To our knowledge, this is the first study to report that NEAT is associated with insulin sensitivity in patients with type 2 diabetes.

Insulin resistance and/or abdominal obesity induce elevation of blood pressure due to sodium retention, sympathetic over-activity, vasoconstriction and activation of the renin-angiotensin system [18]. Regular exercise training has been reported to induce a moderate antihypertensive effect and aerobic exercise has been also reported to lower blood pressure among obese subjects [23,24]. However, the association of NEAT with blood pressure remains to be elucidated. In this study, although a significant association between blood pressure and the NEAT score was not found in patients without abdominal obesity, the NEAT score was significantly and negatively correlated with both systolic and diastolic blood pressure in patients with abdominal obesity. These results suggest that NEAT may be associated with reduction of blood pressure in type 2 diabetic patients with abdominal obesity.

Smoking is a crucial risk factor for atherosclerotic diseases [14,25,26]. Smoking has been reported to accelerate atherosclerosis due to impairment of the platelet and vascular endothelial functions, oxidative stress, and induction of serum lipids abnormalities [27]. Smoking is associated with elevation of TC and TG and reduction of HDL-C levels [28,29]. In this study, serum HDL-C levels (mean ± SD; 45.1 ± 10.1 mg/dl) in smokers were significantly lower than those (55.9 ± 14.5 mg/dl) in ex- and non-smokers (*P* = 0.01 with a Mann–Whitney U test). In our study, the NEAT score was significantly and negatively correlated with baPWV as the clinical marker for atherosclerosis in current smokers. Physical activity has been reported to improve the vascular endothelial function [30,31] and reduce oxidative stress and low-grade inflammation [32], which may partially explain a negative correlation between the NEAT score and baPWV. However, it remains unknown why this association between the NEAT score and PWV was not found in ex- and non-smokers, which requires further study. To our knowledge, this is the first report to show that NEAT is associated with an improvement in the atherosclerotic marker in current smokers with type 2 diabetes.

Limitations of the study need to be addressed. This is a cross-sectional study, limiting inferences of causality and its direction. There are some confounding factors to adjust such as dietary intake, social status, and other life habits. The NEAT score calculated with the questionnaire is subjective data and may not always represent the true NEAT [33,34]. The possibility of recall bias cannot be denied. To determine the exact NEAT score is difficult and complicated, but it is important to evaluate actual physical activity in daily lives rather than in laboratories [35,36]. The validity of methods for measuring NEAT is still controversial, however, we believe that a questionnaire is one of the most useful and reliable methods of measuring NEAT. We should mention further limitations on our approach, including the small sample size and the reliability of self reported activities. The primary drawback of the study was that a questionnaire was used rather than objective measures. A third factor could easily explain both the status of health and the physical activity levels. It could be that a third factor (for example, genetics, or aerobic capacity) underlies both the increased NEAT and the health variables measured [37-43], and that NEAT itself did not improve metabolic parameters. According to the Organization for Economic Co-operation and Development (OECD) Health Data 2012, Japan has a markedly lower obesity rate than most other countries. Our results may not be consistently observed in other countries. Further studies, preferably with larger numbers of subjects, will be needed in the future.

## Conclusions

In conclusion, this study demonstrated that NEAT is associated with amelioration of insulin sensitivity in all participants, and reduction of waist circumference and elevation in HDL-C in women with type 2 diabetes, and is also to improvement in insulin resistance and blood pressure in diabetic patients with abdominal obesity. Furthermore, our study demonstrated that NEAT is associated with amelioration in PWV as the clinical marker for atherosclerosis in current smokers with type 2 diabetes.

## Competing interests

The authors declare that they have no competing interests.

## Authors’ contributions

All eight authors have substantially contributed to conception and design, acquisition of data or analysis and interpretation of data; drafting the article or revising it critically for important intellectual content; and all authors read and approved the final manuscript.

## Supplementary Material

Additional file 1 Non-Exercise Activity Thermogenesis (NEAT) score. Click here for file
