# Supplementary material for: Correlations of non-exercise activity thermogenesis to metabolic parameters in Japanese patients with type 2 diabetes
Source: Diabetol Metab Syndr. 2013 May 27;5:26. doi: 10.1186/1758-5996-5-26 (PMC3671133; doi:10.1186/1758-5996-5-26)
Supplement: Additional file 1 — Non-Exercise Activity Thermogenesis (NEAT) score. [file 1758-5996-5-26-S1.doc]

**(Supplementary data)**

**Non-Exercise Activity Thermogenesis (NEAT) Score**

3 points are scored when subjects answer "A lot".

2 points are scored when subjects answer "Sometimes".

1 point is scored when subjects answer "Hardly ever" or "No".

In question No.4,

3 points are scored when subjects answer "Faster than most people".

2 points are scored when subjects answer "About the same as other people".

1 point is scored when subjects answer "I get passed by other people".

**Primarily walking physical exertion**

1. Do you walk on the way to work?

A lot (over 30 minutes one way)

Sometimes (30 minutes or less)

Hardly ever (or do not travel to work)

2. Do you use a train or a bus (not including taxis or private cars)?

A lot (almost every day)

Sometimes (once or twice a week)

Hardly ever

3. Do you walk a lot while working?

A lot

Sometimes

Hardly ever (or do not work)

4. Do you walk fast or slow?

Faster than most people

About the same as other people

Get passed by other people

5. Do you use stairs?

A lot

Sometimes

Hardly ever (or cannot)

6. Do you go shopping for food or other daily necessities?

A lot (once or more a day)

Sometimes (once or more a week)

Hardly ever

7. Do you walk to eat out (including lunch, and not including travel by car)?

A lot (twice or more a week)

Sometimes (about once a day)

Hardly ever (“Hardly ever” should be less than once a week, “a lot” and “sometimes” should be 2 – 5 days a week.)

8. Do you take the garbage out?

A lot (4 times or more a week)

Sometimes (about once a week)

No

9. Do you go to concerts, to the theatre or karaoke?

A lot (once or more a week)

Sometimes (about once a month)

Hardly ever

10. Do you play with young children outside?

A lot (once or more a day)

Sometimes (once or more a week)

No (or do not have young children)

11. Do you ever go for a walk (including walking the dog)?

A lot (once or more a day)

Sometimes (once or more a week)

Hardly ever

**Non-primarily walking physical exertion**

12. Do you ever do any light cleaning (such as picking up trash)?

A lot (once or more a day)

Sometimes (once or more a week)

Hardly ever

13. Do you ever do any relatively hard cleaning (such as using a vacuum cleaner, mopping a floor, or dusting)?

A lot (once or more a day)

Sometimes (once or more a week)

Hardly ever

14. Do you ever clean any large objects (such as windows, ventilation fans, or cars)?

A lot (once or more a week)

Sometimes (once or more a month)

Hardly ever

15. Do you ever prepare meals (cooking or serving)?

A lot (once or more a day)

Sometimes (once or more a week)

Hardly ever

16. During meals, do you often get up to fill rice bowls, or get things?

A lot

Sometimes

Hardly ever

17. Do you clear the table?

A lot (once or more a day)

Sometimes (once or more a week)

Hardly ever

18. Do you wash the dishes?

A lot (once or more a day)

Sometimes (once or more a week)

Hardly ever

19. Do you wipe the dishes and put them away?

A lot (once or more a day)

Sometimes (once or more a week)

Hardly ever

20. Do you do the washing (carrying and putting out to dry)?

A lot (once or more a day)

Sometimes (once or more a week)

Hardly ever

21. Do you bring the washing in (and fold it up)?

A lot (once or more a day)

Sometimes (once or more a week)

Hardly ever

22. Do you wash the bed sheets and covers?

A lot (every day)

Sometimes (once or more a week)

Hardly ever

23. Do you ever put the bedding outside in the sun?

A lot (once or more a day)

Sometimes (once or more a week)

Hardly ever

24. Do you do the ironing?

A lot (once or more a day)

Sometimes (once or more a week)

Hardly ever

25. Do you clean the bath and the toilet?

A lot (once or more a day)

Sometimes (once or more a week)

Hardly ever

26. Do you clean the garden and around the house?

A lot (once or more a day)

Sometimes (once or more a week)

Hardly ever

27. Do you ever do any weeding or gardening?

A lot (once or more a day)

Sometimes (once or more a week)

Hardly ever

28. Do you water any plants?

A lot (once or more a day)

Sometimes (once or more a week)

Hardly ever

29. Do you look after or feed any pets?

A lot (once or more a day)

Sometimes (once or more a week)

Hardly ever

30. Do you have to look after any young children (such as cooking meals, dressing, or playing together inside)?

A lot (once or more a day)

Sometimes (once or more a week)

Hardly ever

31. Do you ever pick them up (such as giving them a hug or a piggy-back ride)?

A lot (once or more a day)

Sometimes (once or more a week)

Hardly ever

32. Do you have to look after anyone old or sick?

A lot (once or more a day)

Sometimes (once or more a week)

Hardly ever

33. How often do you take a bath (or shower)?

A lot (once or more a day)

Sometimes (once or more a week)

Hardly ever

34. Do you do any sewing or any other handcraft?

A lot (once or more a day)

Sometimes (once or more a week)

Hardly ever

35. Do you play an instrument?

A lot (once or more a day)

Sometimes (once or more a week)

Hardly ever

36. Do you ride a bicycle?

A lot (once or more a day)

Sometimes (once or more a week)

Hardly ever
